# Supplementary material for: Superwavelength self-healing of spoof surface sonic Airy-Talbot waves
Source: Nat Commun. 2023 Nov 22;14:7633. doi: 10.1038/s41467-023-43379-9 (PMC10665557; doi:10.1038/s41467-023-43379-9)
Supplement: Supplementary file 1 — Supplementary Information [file 41467_2023_43379_MOESM1_ESM.pdf]

Supplementary Information for

Superwavelength Self-Healing of Spoof Surface Sonic Airy-Talbot Waves

Hao-xiang Li[1,2*], Jing-jing Liu[1*], Zhao-xian Chen[1], Kai Wu[1], Bin Liang[1†], Jing Yang[1†], Jian-chun Cheng[1†], and Johan Christensen[3†]

[1]*Collaborative Innovation Center of Advanced Microstructures and Key Laboratory of Modern Acoustics, MOE, Institute of Acoustics, Department of Physics, Nanjing University, Nanjing 210093, People's Republic of China*

[2]*College of Information Science and Technology, Nanjing Forestry University, Nanjing 210037, People's Republic of China*

[3]*IMDEA Materials Institute, Calle Eric Kandel, 2, 28906 Getafe, Madrid, Spain*

*These two authors contributed equally to this work.

†Correspondence and requests for materials should be addressed to B.L. (liangbin@nju.edu.cn), J.Y. (yangj@nju.edu.cn), J.C. (jccheng@nju.edu.cn) or J.C. (johan.christensen@imdea.org).

Supplementary Note 1. Field distributions of SSAWs in *y-z* plane.

In contrast to conventional surface acoustic waves that propagate under the surface of solid medium, in our mechanism the produced spoof surface wave is mostly concentrated in the air region with rapidly decaying in the vertical direction, as shown in Supplementary Fig. 1.

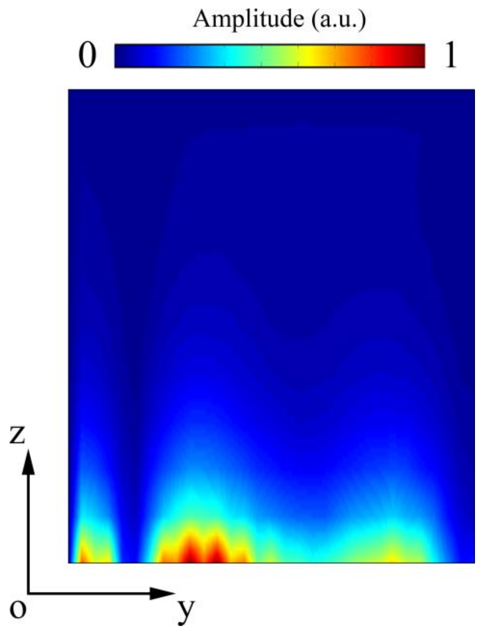

Supplementary Figure 1 | Measured pressure amplitude field of the Airy-Talbot effect at the location of *x*=0.65m in *y-z* plane.

Supplementary Note 2. Equifrequency contours (EFCs) of the SSAW metasurface.

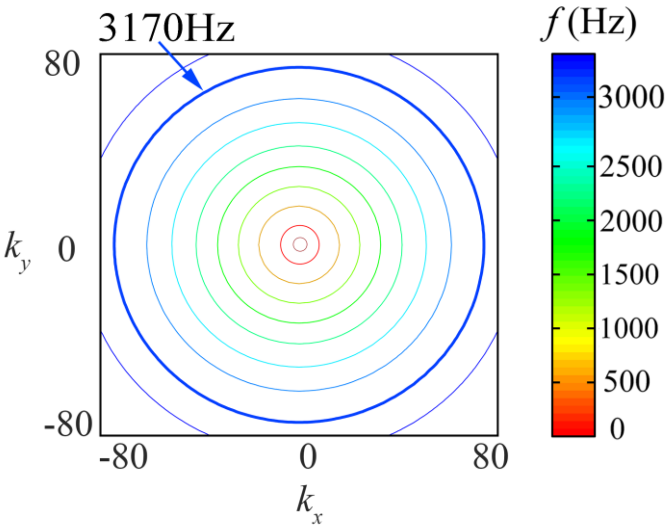

Supplementary Figure 2 | Equifrequency contours (EFCs) of the unit cell. The 3170Hz is marked by the blue line and the circular profile demonstrates the SSAW device can be considered as an isotropic homogeneous material.

At a working frequency of 3170Hz, we operated the holey metasurface at an isotropic regime as the computed equi-frequency contours (EFCs) in Supplementary Fig. 2 displays. Here, the EFC at 3170Hz is near perfectly circular, i.e., $n_{eff} \approx n_X \approx n_M$, which ensures a simplified implementation of the acoustic self-imaging above the isotropic planar surface[1, 2].

Supplementary Note 3. Subwavelength propagation feature of the SSAWs.

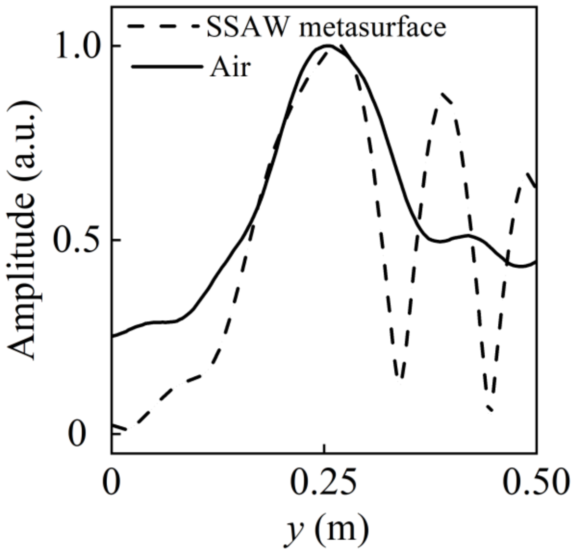

Supplementary Figure 3 | Normalized amplitude distributions of one Airy beam at x = 0.65m. The black dotted and solid lines represent the amplitude profiles of the Airy beams propagating above the SSAW metasurface and in free space, respectively, which show that the main lobe of the Airy beam is narrower comprising multiple side lobes in the former case.

Supplementary Figure 3 shows that the FWHM of the main lobe above the SSAW device is narrower compared to free space, which is caused by the compression of the effective in-plane wavelength of the subwavelength SSAWs. Therefore, unlike highly spread Airy beams in free space comprising larger FWHM of the main lobe, additional side lobes are only seen when the structured metasurface is employed. This feature can also be explained by calculating the characteristic propagation distance of Airy beams, within which the spatial FWHM width of the main lobe can remain almost invariant. It has been theoretically proved the characteristic propagation distance of finite energy Airy beams can be expressed as

follows[3]

$$x_{\max} = 2kb^{-3/2}\sqrt{|y_{\text{cut}}| - |a_1|/b}, \tag{1}$$

where y_{cut} and a_1 represent the position coordinate of the truncation point and the position of the peak of main lobe at the onset of propagation, respectively. k is the wavenumber and b=15m^{-1} is the scale factor. From Eq. (1), we find the characteristic distance depends on several factors, including the beam's initial conditions, such as its width and curvature, as well as the wavelength of the acoustic waves. When $a_1 = 0$, Equation (1) can be simplified as

$$x_{\max} = 2kb^{-3/2}\sqrt{|y_{\text{cut}}|}. \tag{2}$$

Under the same truncation length and operation frequency, the characteristic propagation distance of Airy beams under these two circumstances is solely determined by the wavenumber k. Since the wavenumber above the metasurface $k_{\text{SSAW}} = 78.5\,\text{m}^{-1}$ is significantly larger than that in free space, the characteristic propagation distance of SSAWS is obviously longer, which ensures the subwavelength profiles at the self-imaging plane. We also plot in Supplementary Fig. 4 the dependence of the maximum nondiffracting propagation distance on the position of the truncated-point y_{cut}, from which we can clearly find the $x_{\max}$ of SSAWs is always larger than that in free space under the same source scale. Specifically, we set $y_{\text{cut}} = 0.15\text{m}$ and substitute k_0 and k_{SSAW} into Eq. (2) to calculate the characteristic propagation distances in two circumstances:

$x_{max-air} \approx 0.7\text{m}$ and $x_{max-SSAW} \approx 1\text{m}$. This suggests that for a specific observation position close to $z_{max-air}$ (e.g., x=0.65m), the beam in free space starts to diffract and spread out, while the main and side lobes of SSAW-based Airy beam remain almost invariant due to the longer characteristic distance $z_{max-SSAW}$, demonstrating the significant advantages of SSAWs in terms of subwavelength transmission.

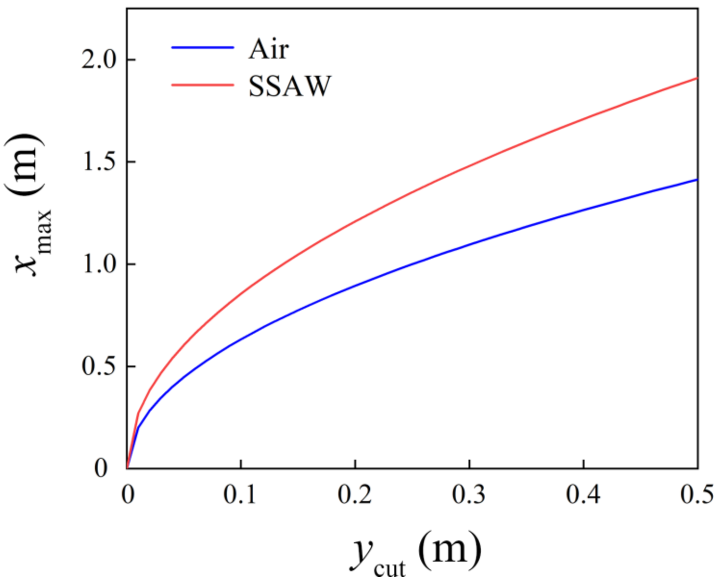

Supplementary Figure 4 | The dependence of the maximum nondiffracting propagation distance of an aperture-truncated Airy beam on the position of the truncated-point y_{cut}. The red and blue solid lines represent the SSAW and the sound waves in free space.

Supplementary Note 4. Comparison of source profiles in air and SSAW metasurface.

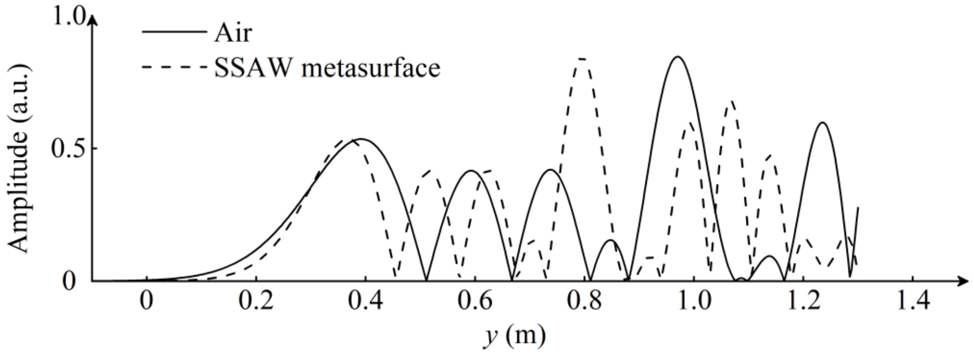

Supplementary Figure 5 | Comparison of the sound source profiles by the superposition of two Airy beams in free space and above the SSAW metasurface at x = 0m. The black dotted and solid lines describe the amplitude profiles where the acoustic waves are transmitted by the SSAW metasurface and air respectively.

Since the distance between two neighboring peaks in the transverse intensity profile is limited to the wavelength of the acoustic Airy beams used[4], the profile can be effectively suppressed by reducing the wavelength. The dispersion curve for the SSAW shows that when the operating frequency is close to the resonance frequency, $k_x \to \infty$ and the equivalent wavelength becomes much smaller than in air, which means that we can transmit acoustic signals beyond the diffraction limit and miniaturize the SSAW system. In order to illustrate this feature quantitatively, we fix the frequency at 3520Hz, which is near the resonance frequency. The

equivalent wave vector of the SSAWs is calculated as $0.62\mathrm{m}^{-1}$, which is three times larger than the one in free space. Therefore, as shown in Supplementary Fig. 5, the spatial profile of the sound source for emitting two Airy beams above the SSAW metasurface can be compressed to nearly 3 times compared to the free-space counterpart.

Supplementary Note 5. Comparison of the diffraction-free property.

In order to observe the self-imaging effect experimentally, the Airy beams should meet the paraxial approximation condition and the spatial distribution of Airy beam needs to satisfy Eq. (3) in the main text, which requires us to quantitatively evaluate the diffraction-free property of Airy beams. By referring to the literature which analyzes the non-diffracting property applying Babinet's principle[5], we calculate the cross correlation between the initial Airy beam's field and the beam's field after propagating same distance in free space and metasurface. It can be seen from the cross-correlation figure below, both beams inevitably suffer from the diffraction effect, while the one above the metasurface maintains its shape better, indicating that we can compress the wavelength to reach for longer Talbot distances, which is consistent with our theoretical and experimental results.

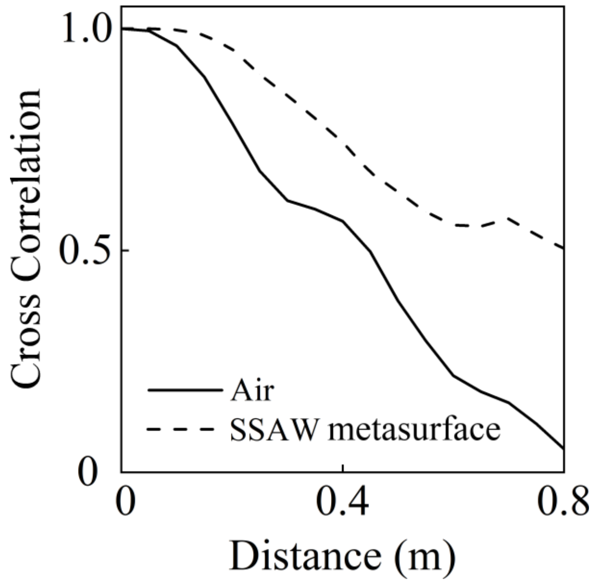

Supplementary Figure 6 | Comparison between the diffraction-free property of Airy beams transmitted by SSAW metasurface and air. Cross correlation describes the similarity between the initial beam's field and propagating beam's field. The black dotted and solid lines represent the SSAW and air, which value is always larger above the metasurface, showing the Airy beams above the SSAW metasurface have a stronger diffraction-free ability.

Supplementary Note 6. Modulation of the Talbot distance with the metasurface.

According to Eq. (11), we can further derive the relation between x_{T}' and λ_{eff} by setting $k_{\mathrm{eff}} = 2\pi/\lambda_{\mathrm{eff}}$

$$x_{\mathrm{T}}' = \frac{4\pi k_{\mathrm{eff}}}{b^3\Delta'} = \frac{8\pi^2}{b^3\Delta'\lambda_{\mathrm{eff}}}, \tag{3}$$

which shows the self-imaging distance is inversely proportional to the wavelength and implies that we can increase or reduce the equivalent wavelength by designing the unit cells of the metasurface to change the Talbot distance, instead of controlling the separation of the Airy beams. To verify this, we plot two acoustic fields above the metasurfaces with different structural parameters, as shown in Supplementary Figs. 7b and c. According to the dispersion relation, we can calculate the wave vectors of SSAWs under these two circumstances as 60m^{-1} and 80m^{-1}, respectively. According to Eq. (3), we theoretically calculate the self-imaging distances approximately as 0.5m and 0.8m under the same source distribution, which are consistent with the simulated results, demonstrating the capability of metasurfaces in modulating Talbot distances.

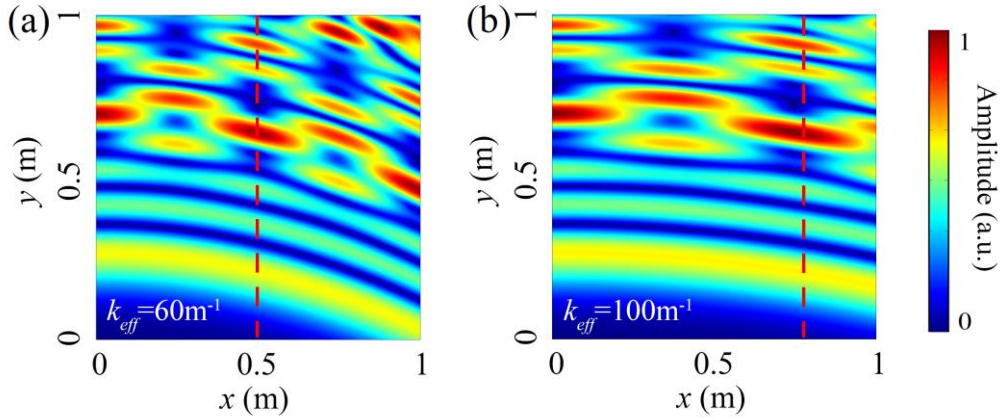

Supplementary Figure 7 | Sound field plots of the Airy-Talbot effect with different wavenumber **a** $k_{\mathrm{eff}} = 60\mathrm{m}^{-1}$ (the diameter of aperture *D* of the resonator is 0.3cm) and **b** $k_{\mathrm{eff}} = 100\mathrm{m}^{-1}$ (the diameter of aperture *D* of the resonator is 0.64 cm). The first Talbot distance is marked by the red dotted lines in **a** and **b**.

Because of the compressed wavelength associated to the band-flattening effect, the Talbot distance in our system will increase as governed by Eq. (3), which is depicted in Supplementary Fig. 8 by virtue of an extended Talbot distance compared to free space. In addition, the sound energy is mainly concentrated above the metasurface via the local-resonance-controlled SSAWs and does not leak into free space, which opens up possibilities for manipulating the wave characteristics for specific applications, such as waveguiding.

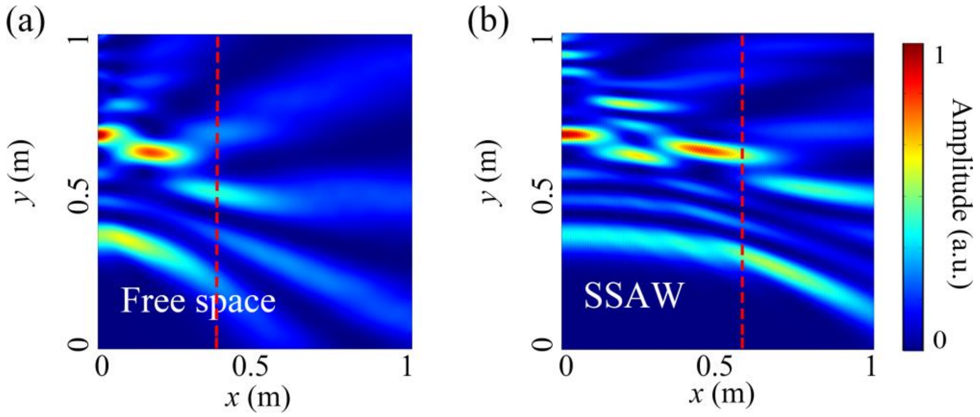

Supplementary Figure 8 | Sound field plots of the Airy-Talbot effect in **a** free space and **b** above the SSAW metasurface. The Talbot distance is marked by the red dotted lines. The simulation is carried out by using the method of effective parameter.

Supplementary Note 7. Proof of the self-imaging effect and calculation of the Talbot distance.

Equation (4) in the main text describes the sound field from superimposed Airy beams that can be simplified as

$$\begin{aligned}|\phi(x,y)| &= \left|\left\{\sum_n c_n \mathrm{Ai}\left[y-\left(\frac{x}{2}\right)^2-n\Delta\right]\exp\left(-\frac{1}{2}in\Delta x\right)\right\}\exp\left[i\left(\frac{xy}{2}\right)-i\frac{x^3}{12}\right]\right| \\ &= \left|\left\{\sum_n c_n \mathrm{Ai}\left[y-\left(\frac{x}{2}\right)^2-n\Delta\right]\exp\left(-\frac{1}{2}in\Delta x\right)\right\}\right|\times\left|\exp\left[i\left(\frac{xy}{2}\right)-i\frac{x^3}{12}\right]\right| \\ &= \left|\left\{\sum_n c_n \mathrm{Ai}\left[y-\left(\frac{x}{2}\right)^2-n\Delta\right]\exp\left(-\frac{1}{2}in\Delta x\right)\right\}\right|,\end{aligned} \tag{4}$$

from which we can see that the change of the amplitude is caused by the $\exp(-in\Delta x/2)$ term. x, y and Δ are the dimensionless coordinate variables and the interval period, respectively. When $x = 4\pi/\Delta$, the field amplitude becomes

$$\left|\phi\left(\frac{4\pi}{\Delta},y\right)\right| = \left|\left\{\sum_n c_n \mathrm{Ai}\left[y-\left(\frac{2\pi}{\Delta}\right)^2-n\Delta\right]\exp(-in2\pi)\right\}\right| = \left|\sum_n c_n \mathrm{Ai}\left[y-\left(\frac{2\pi}{\Delta}\right)^2-n\Delta\right]\right|. \tag{5}$$

Compared with the amplitude distribution at the sound source

$$|\phi(0,y)| = \left|\sum_n c_n \mathrm{Ai}(y-n\Delta)\right|, \tag{6}$$

we find mathematically that the Airy-Talbot effect is equivalent to a $(2\pi/\Delta)^2$ transversely shifted field distribution compared to the source plane.

In the following, we calculate the Talbot distance. Since the calculated equi-frequency contour (EFC) at the working frequency is near perfectly circular, the effective wavenumber can be written as

$$k = k_{\mathrm{eff}} = k_x = k_y = \frac{2\pi}{\lambda_{\mathrm{eff}}}, \tag{7}$$

where k_{eff} and λ_{eff} are the effective wavenumber and wavelength. Considering that Eq. (3) is the solution of the paraxial wave equation, i.e.,

$$i\partial_x \phi + \frac{1}{2}\partial_y^2 \phi = 0, \tag{8}$$

the coordinate variables x, y and the interval period of Airy beams Δ in Eqs. (3)-(5) need to be dimensionless

$$x = \frac{b^2 x'}{k_{\mathrm{eff}}}, \quad y = by', \quad \Delta = b\Delta', \tag{9}$$

where x', y' and Δ' represent the real-space coordinate variables and interval period, respectively. b=15m^{-1} is the scale factor. With the Talbot distance written as

$$x_{\mathrm{T}} = \frac{4\pi}{\Delta}, \tag{10}$$

and substituting Eq. (9) into (10), we obtain the expression for the self-imaging distance in real coordinates as

$$x_{\mathrm{T}}' = \frac{k_{\mathrm{eff}} x_{\mathrm{T}}}{b^2} = \frac{4\pi k_{\mathrm{eff}}}{b^3 \Delta'}. \tag{11}$$

Considering the interval of Airy beams Δ' is 0.45m, the self-imaging distance amounts to approximately 0.65m.

Supplementary Note 8. Validity of the proposed method by using two Airy beams.

As we know, more incident Airy beams will allow a better Talbot effect and more communication channels, which, however, will call for larger experimental space and more complicated design of multi-drivers. In our current design, in order to facilitate the experimental implementation, we emit two Airy beams to display the Airy-Talbot effect, which is sufficient to validate the mechanism of this self-imaging effect and can significantly reduce the size of the sound source. More detailed discussion is explained in the following:

Firstly, Supplementary Figures 9d-f show that as more Airy beams are superimposed, the amplitude profile with more subwavelength details appears where the narrowest width of each lobe on the right is nearly 3cm, making the desired discrete amplitude profile hard to realize with the 1.3-in. loudspeakers used in our experiment. Considering this technical issue, we used only two Airy beams to explore this effect.

In addition, we depict the sound amplitude plots of the Airy-Talbot effect by the superposition of 2, 3 and 10 Airy beams, as shown in Supplementary Figs. 9a-c, from which we find more communication channels can be built when we set the launch point as y=0m, y=0.45m, y=0.9m, etc. However, considering the solutions of the Airy function change from oscillatory to exponential attenuation at the turning point, the nth Airy beams

(n>2) hardly affects the interference sound field of the first two Airy beams. Supplementary Figures 9d-f show the amplitude profiles of the first self-imaging plane, as marked by red dotted lines in a-c, from which we can find the amplitude profile by the superposition of the first two Airy beams (y<0.8m) maintains almost unchanged in all three conditions, implying that we can explore this effect with two Airy beams, which is adequate to showcase the Airy-Talbot's mechanism.

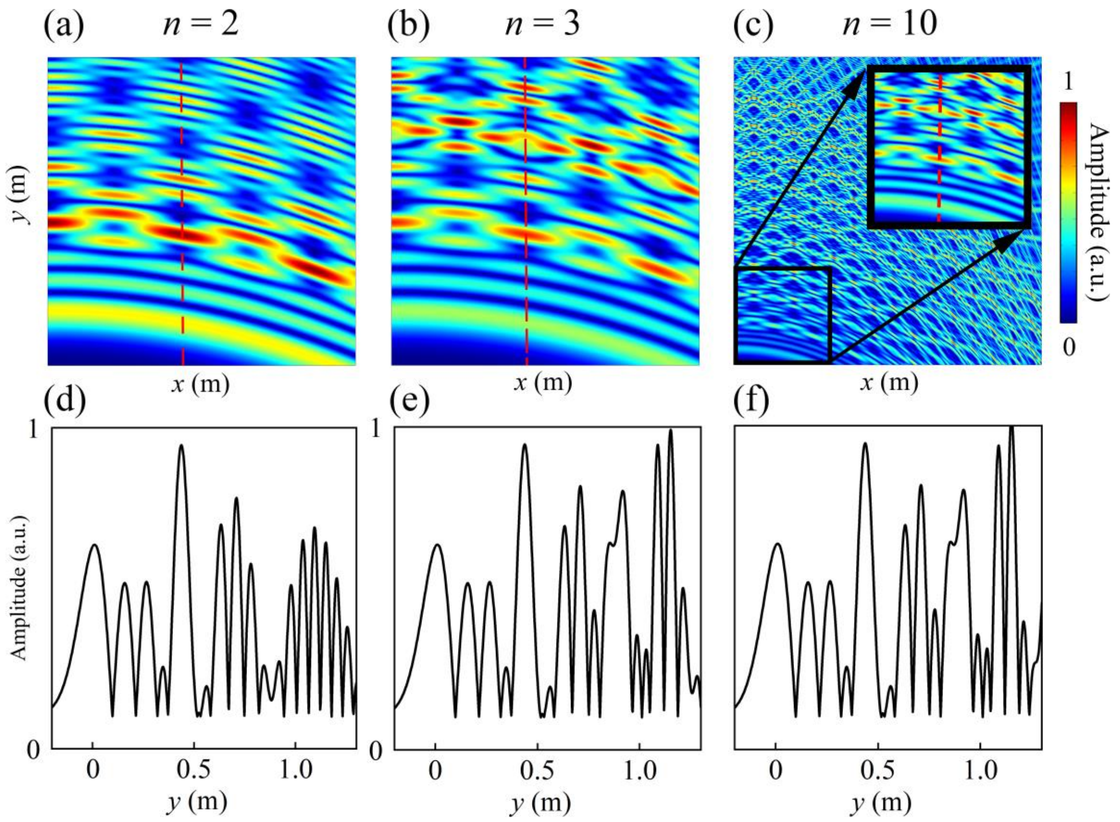

Supplementary Figure 9 | Pressure amplitude plots of transmitting **a** n=2, **b** n=3 and **c** n=10 Airy beams. The upper-right inset shows the zoomed-in view of the black box in **c**. Pressure amplitude profiles at the first self-imaging plane of transmitting **d** n=2, **e** n=3, and **f** n=10 Airy beams, which are marked by the red dotted lines in **a-c**.

Supplementary Note 9. Effect of the thermoviscous losses.

In the Helmholtz resonators that we designed, the thermoviscous losses typically occur in the narrow regions, which may cause the attenuation of SSAWs and deserve further consideration in practice. In order to evaluate the influence of the losses on the SSAWs, we calculate the SSAW dispersion relation for a Helmholtz cavity with a narrow neck $r = 0.1\times10^{-2}\,\mathrm{m}$ [6]

$$\frac{i\omega\rho_0}{\sqrt{k_x^2-k_0^2}}=\frac{a^2}{\pi\left(D/2\right)^2}\left(\rho_0 c_0\frac{2i\sin\left(k_c d/2\right)}{\sqrt{\left(\gamma-(\gamma-1)\Psi_h\right)\Psi_v}}+\frac{i\pi\left(D/2\right)^2}{\omega C_{\mathrm{HR}}}\right), \tag{12}$$

where k_x is the wavenumber of the SSAWs, k_0 is the wavenumber in the free space, ω is the angular frequency, $\rho_0 = 1.21\,\mathrm{kg/m^3}$ is the density of air, $c_0 = 343\,\mathrm{m/s}$ is the sound velocity in the air, C_{HR} is the acoustic mass and the acoustic capacitance of HRs, $\mu = 1.85\times10^{-5}\,\mathrm{kg/(m\cdot s)}$ is the dynamic viscosity of air, $\kappa = 0.0258\,\mathrm{W/(m\cdot K)}$ is the fluid thermal conductivity, $c_{P0} = 1.005\times10^{3}\,\mathrm{J/(kg\cdot K)}$ is the specific heat at constant pressure and $\gamma = 1.4$ is the ratio of the specific heat at constant pressure and constant volume. We introduce the viscous wave number k_v and the thermal wave number k_h as

$$k_v^2=-i\omega\frac{\rho_0}{\mu},\quad k_h^2=-i\omega\frac{\rho_0 c_{P0}}{\kappa}. \tag{13}$$

Then the function of viscous and thermal fields can be, respectively, derived as

$$\Psi_v = \frac{J_2(k_v D/2)}{J_0(k_v D/2)}, \quad \Psi_h = \frac{J_2(k_h D/2)}{J_0(k_h D/2)}, \tag{14}$$

where J_n is the Bessel function of the first kind and order n. Then the complex wave number can be calculated as

$$k_c^2 = k_0^2 \left(\frac{\gamma - (\gamma - 1)\Psi_h}{\Psi_v} \right). \tag{15}$$

We have calculated in Supplementary Fig. 10 the dispersion curve, which illustrates that the real part of the wavenumber of the SSAWs gradually deviates from the lossless dispersion curve while the imaginary part progressively increases as the frequency rises, showing an obvious attenuation along the wave propagation direction. Consequently, we choose a larger diameter of the aperture D = 0.7cm and a relatively low operating frequency f = 3170Hz to alleviate the thermoviscous effects. Actually, since the thicknesses of the viscous and thermal boundary layers at the operation frequency can be calculated as

$$d_\mu = \sqrt{\frac{2\mu}{\omega\rho_0}} \approx 3.9\times10^{-5}\text{m}, \quad d_\kappa = \sqrt{\frac{2\kappa}{\rho_0 c_{P0}\omega}} \approx 4.6\times10^{-5}\text{m}, \tag{16}$$

whose value is much smaller than the radius of the neck, the attenuation of SSAWs caused by the thermoviscous losses can be restricted to a relatively low level[7], which ensures the self-imaging effect even over an extended distance. To be specific, we calculate the equivalent refractive index nearly as 1.42- 0.02i, which shows the inherent losses only slightly change the index values and the attenuation is negligible.

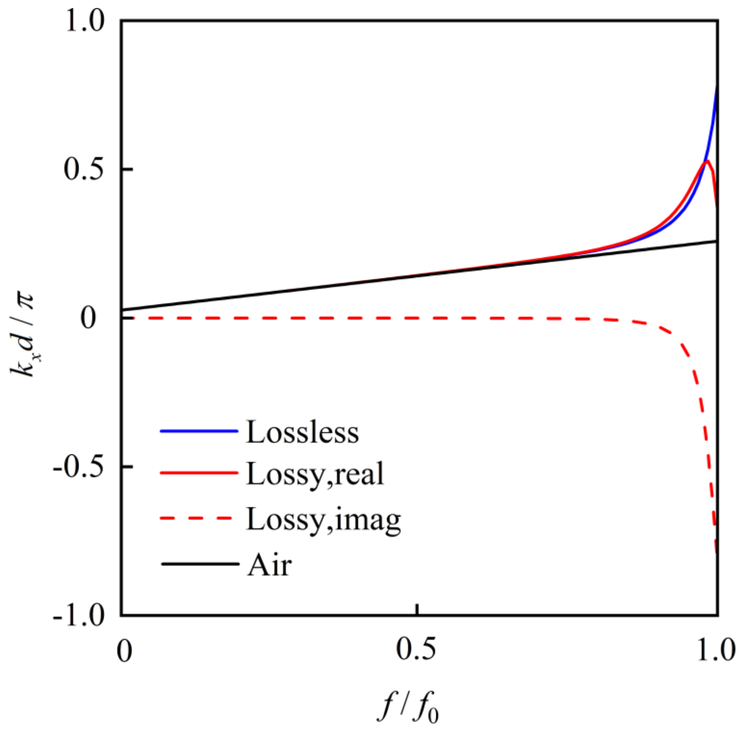

Supplementary Figure 10 | Calculated dispersion relation of the SSAWs in the presence of the thermal and viscous losses. For the lossy case, the wave vector of the SSAWs becomes a complex number (red solid line: real part; red dashed line: imaginary part). The curves of the lossless case (blue solid line) and the air line (black solid line) are also presented for comparison. Here $f_0 = \sqrt{1/M_{\mathrm{HR}} C_{\mathrm{HR}}} / 2\pi$ is the cavity resonance frequency. The unit cell is designed to have $a = 2 \times 10^{-2}\,\mathrm{m}$, $t = 0.75 \times 10^{-2}\,\mathrm{m}$, $D = 0.2 \times 10^{-2}\,\mathrm{m}$ and $d = 0.1 \times 10^{-2}\,\mathrm{m}$.

Supplementary Note 10. Influence of the source's length on the Airy-Talbot effect.

To explore the influence of the source's length on the Airy-Talbot effect, we depict the sound amplitude plots of the Airy-Talbot effect using sound sources of different lengths, as shown in Supplementary Figs. 11a-c, from which we can find as the length decreases, the value of the characteristic propagation distance $x_{\max}$ becomes smaller, resulting in lower periodic repetitions. To be specific, although the amplitude profiles at two self-imaging planes are similar in our current experiment, the central peak at the second Talbot distance gradually shifts from its original position and the value becomes smaller than the lobe's on its left, as shown in Supplementary Figs. 11e and f, revealing that the self-imaging effect can only take place for a few times and will disappear with the propagation of SSAWs. However, by extending the length of the source, the periodic repetition could happen for more times.

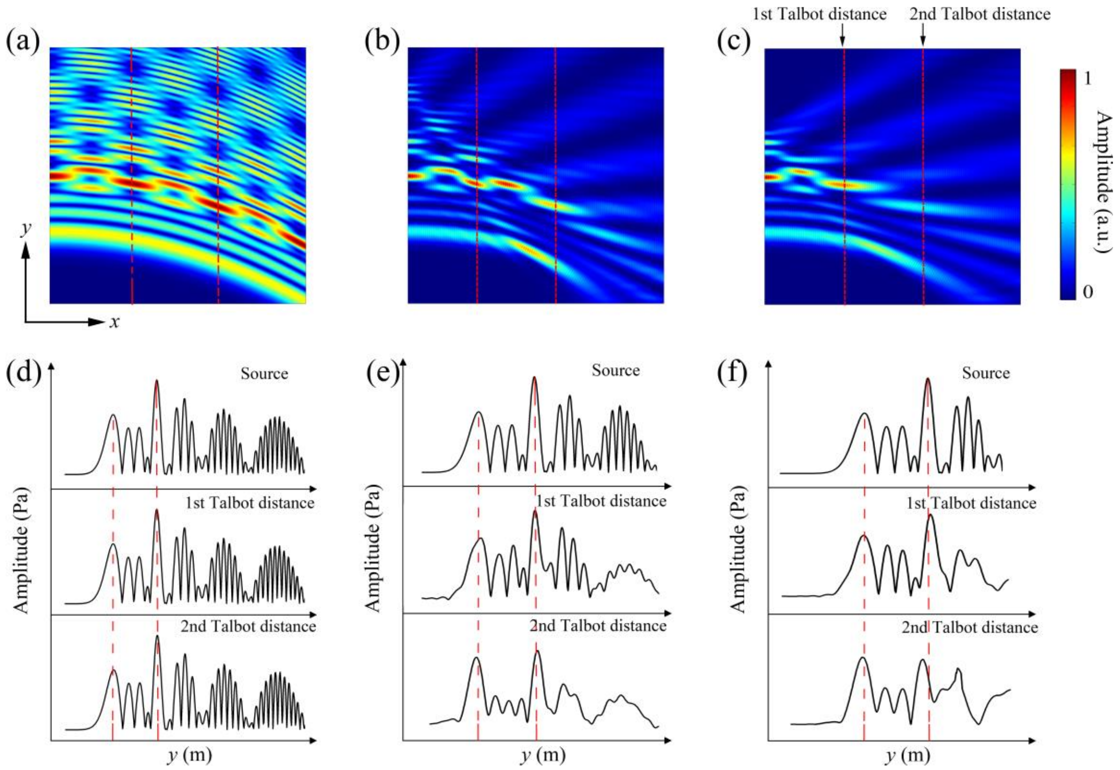

Supplementary Figure 11 | Pressure amplitude plots of transmitting **a** two infinite energy Airy beams and finite energy Airy beams with **b** the position of the truncated-point y_{cut}=1.5m and **c** y_{cut} =1m, where the first and second Talbot distances are marked by the red dotted lines. **d**-**f** Pressure amplitude plots and profiles at the sound source and two self-imaging planes denoted by red dotted lines in **a**-**c**, respectively.

Supplementary Note 11. Experimental verification of robust self-healing.

We insert a rigid cylinder with the radius of 1.3cm/2.5cm/5.1cm/7.6cm/11.2cm/12.7cm into the curved path to block the main lobe of the beam. Supplementary Figure 12 represents the simulated and experimental results about the acoustic field and the amplitude distribution on the self-imaging plane, respectively. We can find that as long as the radius of the scatterer does not exceed 11.2cm (ka=8.8), the normalized amplitude at y=0.43m formed by the interference of different Airy beams will maintain 1 and the similar amplitude profile as the source's can be measured, demonstrating the robustness to scattering media even along a curved trajectory.

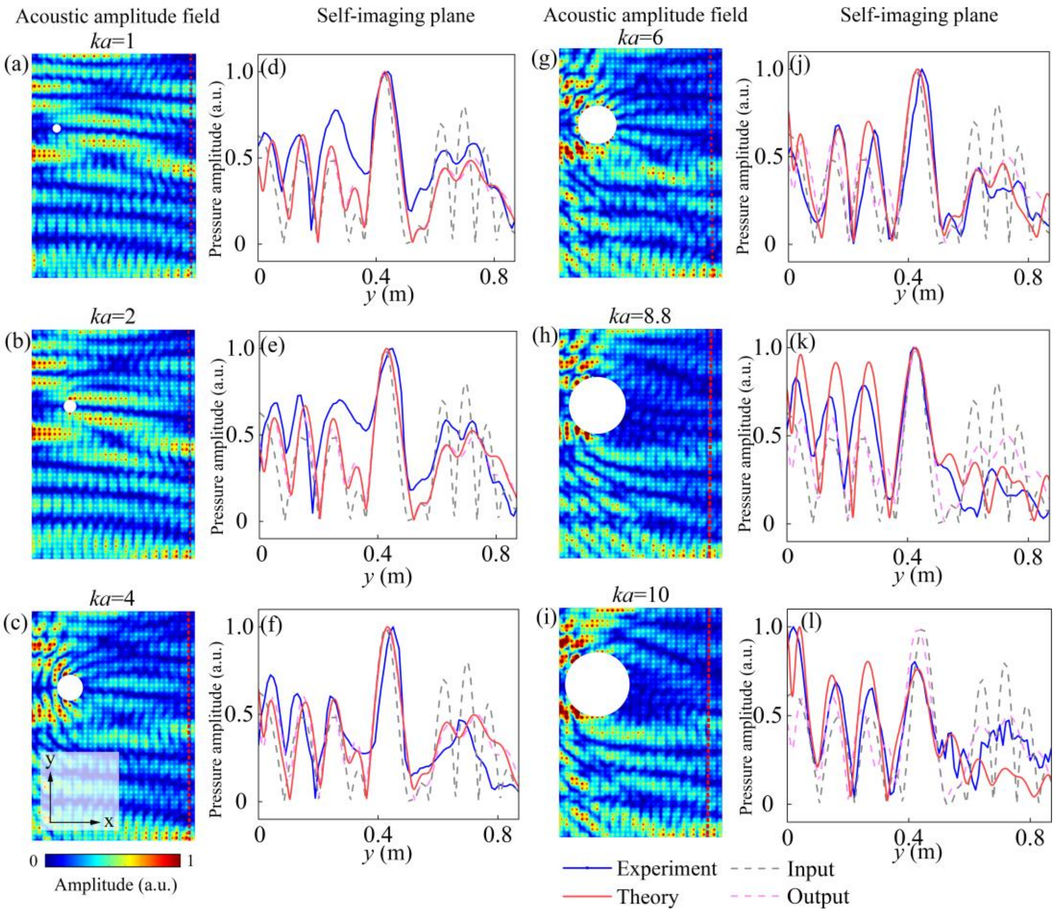

Supplementary Figure 12 | Demonstration of self-construction feature of Airy-Talbot effect. Scatterers with different radii (**a** *ka*=1, *a*=1.3cm **b** *ka*=2, *a*=2.5cm **c** *ka*=4, *a*=5.1cm **g** *ka*=6, *a*=7.6cm **h** *ka*=8.8, *a*=11.2cm **i** *ka*=10, *a*=12.7cm) are located at (*x*=0.15m, *y*=0.6m), respectively. *k* is the equivalent wave number and *a* is the radius of the obstacle. **d**-**f** and **j**-**l** Amplitude distributions along the self-imaging plane, which are denoted by red dashed lines in Supplementary Figs. 12 **a**-**c** and **g**-**i**. The theoretical, experimental results, input and output signals (without the object) are denoted by the red, blue, grey and pink lines, respectively.

In addition, we add a cylinder to quantitatively analyze the influence of scattering cross section on the self-imaging effect. Two cylinders with the same size are located in (*x*=0.15m, *y*=0.3m) and (*x*=0.15m, *y*=0.6m),

respectively, as shown in Supplementary Figs. 13a and b, demonstrating that as long as $ka \leq 6$, a relatively strong correlation is shared by the input and output signals and a robust transmission can be ensured.

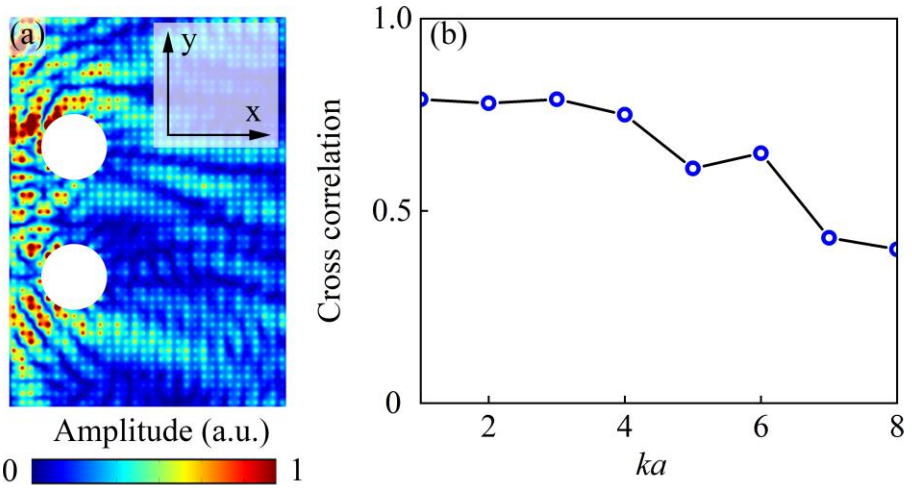

Supplementary Figure 13 | a Simulated acoustic fields with two cylinders (*ka*=6) placed at (*x*=0.15m, *y*=0.3m) and (*x*=0.15m, *y*=0.6m), respectively. **b** Comparison between the input and output signals with two cylinders (*ka*=1, 2, 3, 4, 5, 6, 7, 8).

Supplementary Note 12. Experimental measurement of the acoustic field.

In order to demonstrate the self-healing feature of Airy-Talbot effect better, the according acoustic field distributions have been measured, as shown in Supplementary Fig. 14. The experimental measurement area is $0.4\text{m} \times 0.8\text{m}$, which is denoted by the red box in the simulated acoustic field. The entire sound field near the metasurface sample is measured utilizing a 1/4-in. free-field microphones (Brüel & Kæjr type-4961) which is attached to a 3D stepping motor to scan the target region point by point. The measured height is selected as 2cm above the metasurface sample for all measurements in the x-y plane. The experimental results demonstrate that the sound field distribution remains almost unchanged despite the presence of the cylinder scatters. In particular, the pronounced pressure peak of the main lobe at the center (y=0.43m) shows the remarkable self-healing property even when the radius of the obstacle reaches ka=8.8 (Supplementary Fig. 14l), which reveals the high-quality robust data transmission and would be highly desired for diverse fields ranging from underwater to on-chip communications.

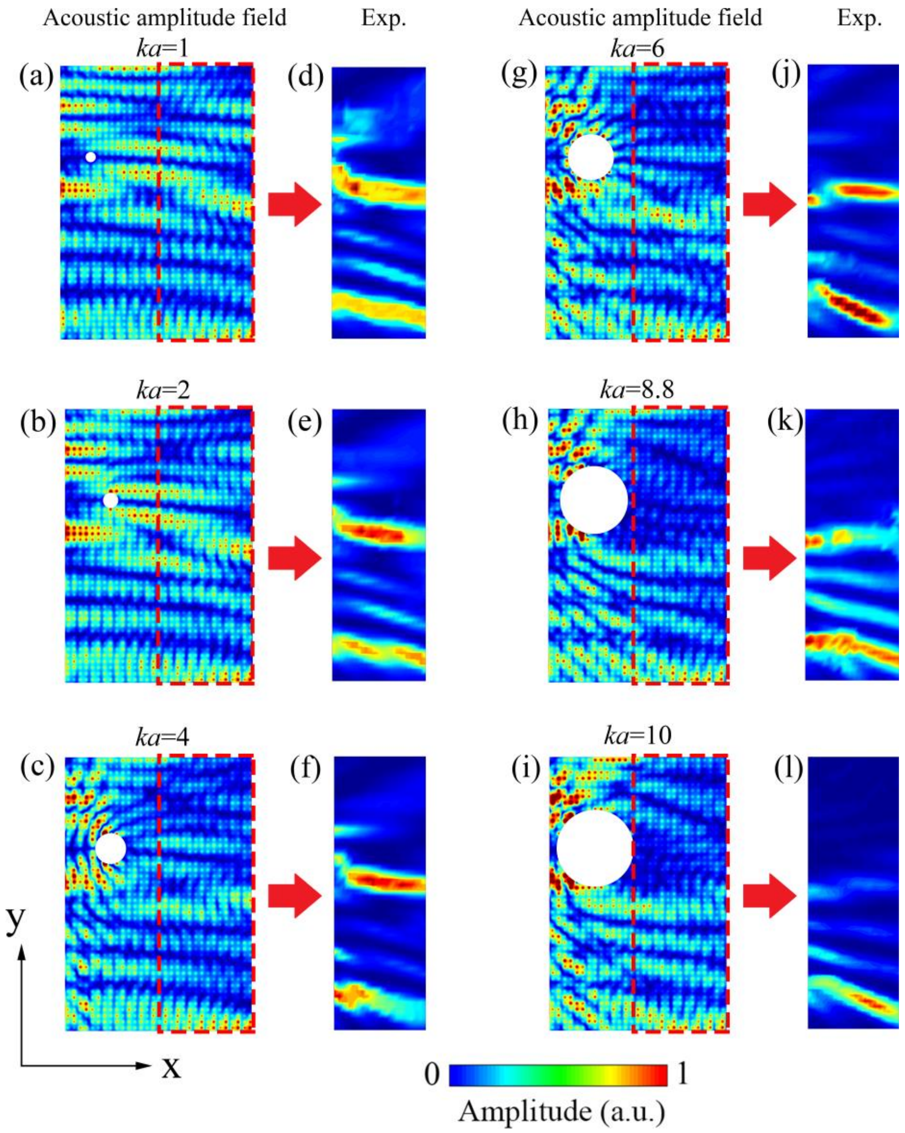

Supplementary Figure 14 | Experimental demonstration of self-healing feature of Airy-Talbot effect. Scatterers with different radii (**a** *ka*=1, *a*=1.3cm **b** *ka*=2, *a*=2.5cm **c** *ka*=4, *a*=5.1cm **g** *ka*=6, *a*=7.6cm **h** *ka*=8.8, *a*=11.2cm **i** *ka*=10, *a*=12.7cm) are located at (*x*=0.15m, *y*=0.6m), respectively. *k* is the equivalent wave number and *a* is the radius of the obstacle. **d-f** and **j-l** Experimentally measured amplitude fields for the area marked within the red box denoted in **a-c** and **g-i**.

Supplementary Note 13. Comparison between the Airy-Talbot effect and the conventional Talbot effect.

We compare the Airy-Talbot effect with the conventional Talbot effect in facing the same obstruction (*ka*=8.8), as shown in Supplementary Figs. 15b and c. From the figures, within the same Talbot distance, the periodic acoustic field of conventional Talbot effect is scattered obviously by the cylinder and cannot replicate itself. We also calculate the cross-correlation coefficient, as denoted by the red hollow squares in Supplementary Fig. 15a, which value is obviously lower than that with Airy beams (blue hollow circles).

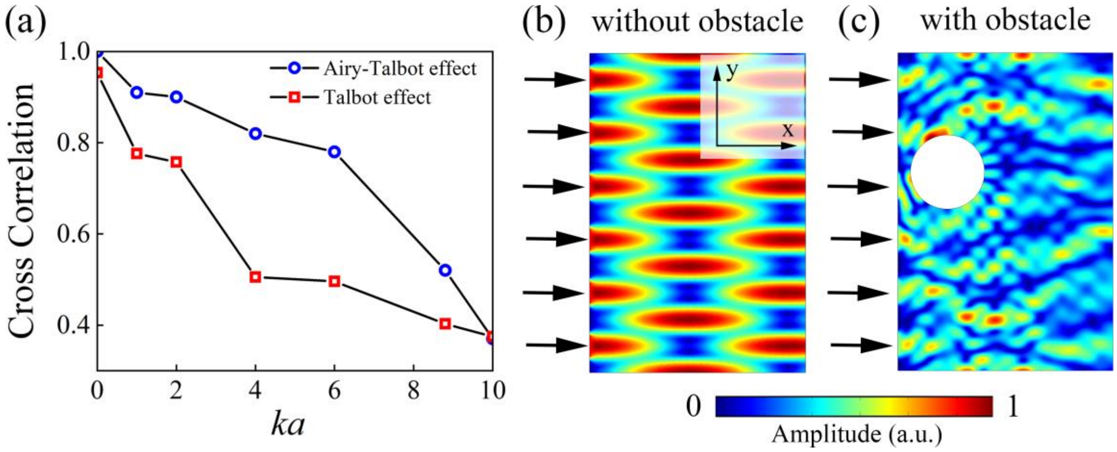

Supplementary Figure 15 | Comparison between the Airy-Talbot effect and conventional Talbot effect. a Comparison between the output and input signals with cylinders (*ka*=1, 4, 8.8 (upper limit), 10) placed at (*x*=0.15m, *y*=0.6m) above the designed SSAW device. The Airy-Talbot effect and conventional Talbot effect are denoted by the blue hollow circles and red hollow squares, respectively. Simulated acoustic fields of the conventional Talbot effect **b** without the cylinder and **c** with the cylinder (*ka*=8.8). Black arrows on

the left represent the incident waves.

Supplementary Note 14. Parallel transmission based on the Airy-Talbot effect.

Here, we validate the function of signal transmission of this SSAW device. By using the existence of the two peaks at y=0m and y=0.43m at the self-imaging plane shown in Supplementary Fig. 16e, we can realize the parallel transmission of coding sequences. We set the positions of (x=0.65m, y=0m) and (x=0.65m, y=0.43m) as the observation points. The offset of the y-coordinate is caused by the curved path shown in Supplementary Figs. 16a and f. To intuitively validate the function of acoustic communication of our SSAW device, the experimentally measured amplitude plots in the square area above the metasurface are shown in Supplementary Figs. 16b and g. The scanning height is controlled by adjusting the vertical position of the microphone, where the measured height is selected as 2cm above the metasurface sample for all measurements in the x-y plane. Based on the transmission principle above, we find out obviously from Supplementary Figs. 16e and j that two different signals {1,1} {0,1} are detected accurately on the receiving plane. For the signal {1,1}, two peaks are clearly measured on the measuring line. For the signal {0,1}, the normalized sound pressure amplitude about 1 Pa

is received at y=0.5m, while nearly no sound pressure signal is measured at y=0m.

The problem of signal crosstalk is also discussed here. We find that when we transmit {1,0}, 0.32Pa is measured at (x=0.65m, y=0.43m), and then the crosstalk is -4.2dB if we define the crosstalk as $10\log(P_1/P_2)$, where P_1 is the sound pressure measured when only one channel is open and P_2 is the sound pressure measured when both channels are open. When the coding sequence {0,1} are transmitted, any sound signals can hardly be detected at (x=0.65m, y=0m). Compared with the previous works of multiplexing [2, 8, 9], it shows the SSAW device may provide good crosstalk isolation.

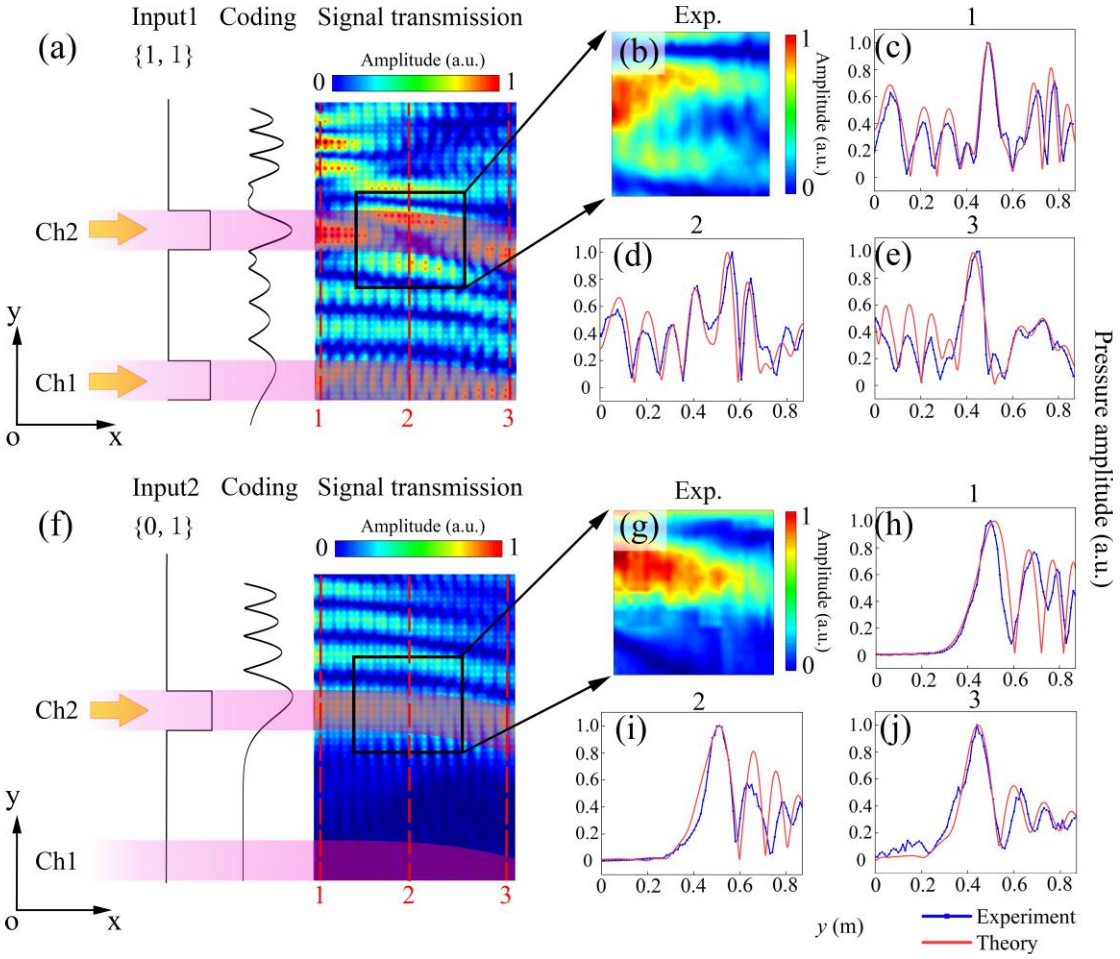

Supplementary Figure 16 | Digital coding and parallel transmission from different source sequences. a and **f** Schematic diagrams for transmitting 2-bits coding sequences: **a** {1,1}, **f** {0,1}. The data using two channels (Ch1/Ch2) are encoded by Airy beams and then transmit along the curved path marked in purple. **b** and **g** Experimentally measured amplitude fields for the area marked within the box denoted in **a** and **f**. **c-e** and **h-j** Normalized amplitude profiles along three planes: x_1 = 0.05m, x_2 = 0.25m and x_3 = 0.65m, marked by red dashed lines in **a** and **f**, respectively.

Supplementary Note 15. Extracting the data stream from time-domain signal with cross-correlation method.

To sufficiently prove the effectiveness of our proposed mechanism, we show the parallel transmission of two 50-bit data streams from the two

binary images, as illustrated in Supplementary Fig. 17. In our implementation, two independent channels, marked as Ch1 to Ch2, are utilized to transmit two-path synthesized Airy beams, with which we could encode two data streams into the amplitude of each Airy beams in 2ASK format. The time-domain signals received at two measurement points defined in the manuscript are also plotted in Supplementary Fig. 17a, corresponding to the two data streams respectively. For the purpose of extracting data streams from the time-domain signals, we perform the cross correlation by multiplying the received signals with an ideal sinusoidal signal with frequency of 3170 Hz to extract the transmission data from time-domain signals. The results of correlation spectra are shown in Supplementary Fig. 17b, where the length of the sinusoidal signal is equal to the length of one pulse period of the received signal. We use a threshold of 0.5 to determine that the transmitted information is “1” or “0”. The decoded data streams in two channels are plotted in Supplementary Fig. 17a (marked by dots) in comparison with the target signal. Obviously, the decoded information streams are consistent well with the input ones without any distortion, indicating that our proposed mechanism allows to achieve real-time and high-precision decoding of information transmitted over two independent channels even in the absence of complicated postprocessing indispensable for conventional mechanisms.

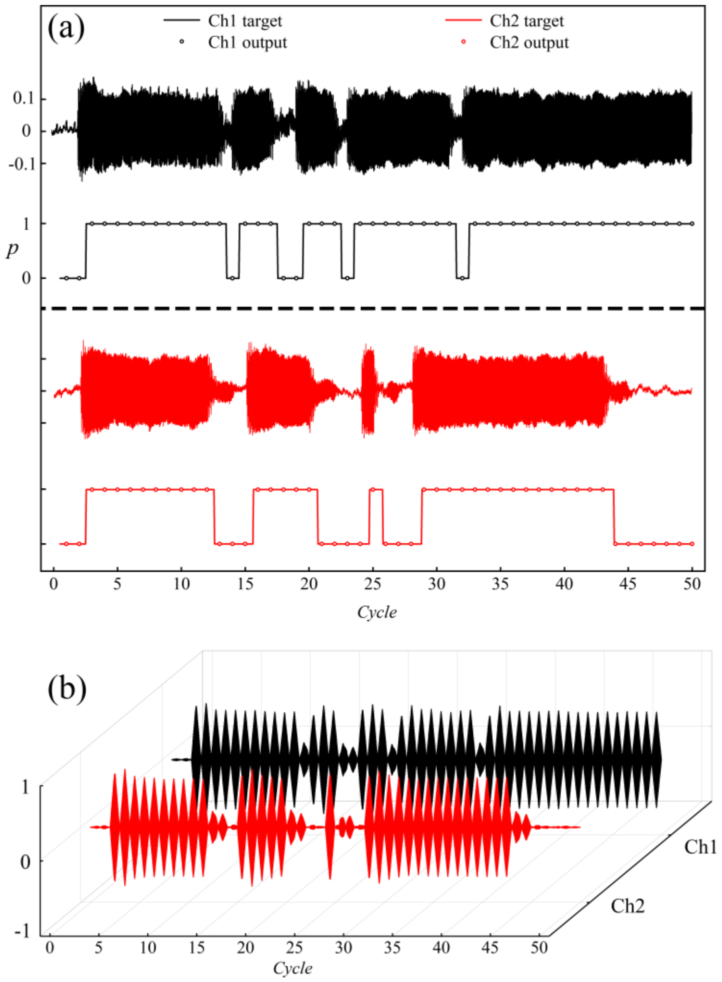

Supplementary Figure 17 | a Real-time communication of two 50-bit data streams in parallel. The comparison between the target data stream and the received output in the two channels, and the measured time-domain signals in each pulse period in two channels. **b** The waveforms of correlation function between the time-domain signals received at two points and an ideal sinusoidal signal with the frequency of 3170 Hz.

Supplementary References

1. Liu, T., Chen, F., Liang, S. J., Gao, H. & Zhu, J. Subwavelength sound focusing and imaging via gradient metasurface-enabled spoof surface acoustic wave modulation. *Phys. Rev. Appl.* **11**, 034061 (2019).

2. Li, B. S., *et al.* MIMO-OFDM for high-rate underwater acoustic communications. *IEEE J. Ocean. Eng.* **34**, 634-644 (2009).

3. Chu, X. C., Zhao, S. H. & Fang, Y. W. Maximum nondiffracting propagation distance of aperture-truncated Airy beams. *Opt. Commun.* **414**, 5-9 (2018).

4. Rogel-Salazar, J., Jimenez-Romero, H. A. & Chavez-Cerda, S. Full characterization of Airy beams under physical principles. *Phys. Rev. A* **89**, 023807 (2014).

5. Bouchal, Z., Wagner, J. & Chlup, M. Self-reconstruction of a distorted nondiffracting beam. *Opt. Commun.* **151**, 207-211 (1998).

6. Huang, S., Fang, X. S., Wang, X., Assouar, B., Cheng, Q. & Li, Y. Acoustic perfect absorbers via Helmholtz resonators with embedded apertures. *J. Acous. Soc. Am.* **145**, 254-262 (2019).

7. Lawrence E. K., Austin R. F., Alan B. C. & James V. S., Fundamentals of Acoustics, 4th ed. (J. Wiley & Sons, Inc., New York, 2000), Chap. 10, pp. 272–274.

8. Jiang, X., Liang, B., Cheng, J. C. & Qiu, C. W. Twisted acoustics: metasurface-enabled multiplexing and demultiplexing. *Adv. Mater.* **30**, 1800257 (2018).

9. Singer, A. C., Nelson, J. K. & Kozat, S. S. Signal processing for underwater acoustic communications. *IEEE Commun. Mag.* **47**, 90-96 (2009).
